# Genomic Surveillance of SARS‐CoV‐2 in Senegal (2020–2024): Variant Turnover, Omicron Introductions, and Interregional Spread

**DOI:** 10.1111/irv.70273

**Published:** 2026-06-12

**Authors:** Ndeye Awa Ndiaye, Safiétou Sankhe, Mamadou Malado Jallow, Mouhamed Kane, Khadidiatou Ndour, Amadou Diallo, Maimouna Mbanne, Ndeye Marième Top, Seynabou Mbaye Ba Souna Diop, Madeleine Dieng, Issa Gnasse, Debora Goudiaby, Cheikh Talla, Mamadou Aliou Barry, Oumar Faye, Ousmane Faye, Cheikh Loucoubar, Amadou Alpha Sall, Gamou Fall, Marie Henriette Dior Ndione, Moussa Moïse Diagne, Ndongo Dia

**Affiliations:** ^1^ Virology Department Institut Pasteur de Dakar Dakar Senegal; ^2^ Vaccine Research Center Institut Pasteur de Dakar Dakar Senegal; ^3^ Epidemiology, Clinical Research and Data Science Department Institut Pasteur de Dakar Dakar Senegal; ^4^ Public Health Direction Institut Pasteur de Dakar Dakar Senegal

**Keywords:** genomic surveillance, omicron, phylogeography, SARS‐CoV‐2, Senegal, viral evolution

## Abstract

**Background:**

Genomic surveillance is central to tracking SARS‐CoV‐2 evolution, variant replacement, and transmission dynamics. In Senegal, the Institut Pasteur de Dakar generated one of the largest national longitudinal SARS‐CoV‐2 genomic datasets, offering a unique view of viral spread from the first pandemic wave to the post‐Omicron period.

**Aims:**

This study aimed to reconstruct SARS‐CoV‐2 evolution and dissemination in Senegal from March 2020 to September 2024, assess variant and Omicron sublineage dynamics, infer introduction and interregional spread patterns, and describe the national mutational landscape.

**Material and Methods:**

A total of 4820 SARS‐CoV‐2 genomes generated by the Institut Pasteur de Dakar were analyzed. Lineage assignment, temporal and regional distribution, time‐scaled phylogenetics, discrete phylogeographic reconstruction, and mutation profiling were used to assess variant turnover, international seeding, intranational dissemination, and genomic diversification.

**Results:**

Ancestral A/B lineages predominated in 2020 before replacement by Alpha and Beta in early 2021, followed by Delta in mid‐2021, which coincided with the highest case burden. From late 2021, Omicron became dominant, with rapid turnover of BA.1/BA.2, BA.4/BA.5, BQ.1.1, XBB, and BA.2.86, while later waves were smaller. Dakar contributed 64.7% of genomes and emerged as the main hub for inferred introductions and interregional spread, with Kaolack and Diourbel as secondary hubs. Major Omicron sublineages resulted from multiple introductions, mainly from Africa and Europe, with BA.1/BA.2 seeded from a broader range of continents than later lineages. Mutation profiling showed strong enrichment in Spike, with recurrent changes in ORF1a/ORF1b and nucleocapsid.

**Discussion:**

The findings reveal repeated variant replacement, shifting international seeding routes, and a hub‐and‐spoke pattern of national dissemination centered on Dakar. Declining later waves may reflect increasing hybrid population immunity, although interpretation remains influenced by heterogeneous sequencing intensity.

**Conclusions:**

This study provides an integrated national picture of SARS‐CoV‐2 evolution in Senegal and supports decentralized sequencing, stronger metadata capture, and routine use of genomic evidence for public health decision‐making.

## 1 Introduction

Infectious respiratory diseases are a major global public health challenge and remain among the leading causes of morbidity and mortality worldwide, particularly in low‐ and middle‐income countries [1]. Airborne and contact transmission can enable rapid spread, especially in densely populated urban settings, and this risk is amplified by globalization, increased human mobility, and persistent inequities in access to healthcare. Emerging respiratory viruses therefore pose recurrent threats, as illustrated by SARS‐CoV‐1 in 2003, MERS‐CoV in 2012, and SARS‐CoV‐2 in late 2019 [2].

SARS‐CoV‐2, the etiological agent of coronavirus disease 2019 (COVID‐19), is a positive‐sense single‐stranded RNA virus in the family Coronaviridae, with high evolutionary potential driven by continuous mutation accumulation [3, 4]. First identified in December 2019 in China, SARS‐CoV‐2 rapidly spread worldwide and triggered an unprecedented pandemic in the 21st century. Since the onset of the pandemic, numerous genetically distinct variants have emerged, some carrying mutations, particularly in the Spike protein, associated with increased transmissibility, partial escape from infection‐ or vaccine‐induced immunity, or reduced effectiveness of certain antiviral therapies [5]. These lineages have been classified by the World Health Organization as variants of concern (VOCs), with Alpha, Beta, Delta, and Omicron driving successive global waves of transmission [6].

In this context of rapid viral evolution, genomic surveillance has become essential to monitor the emergence, diversification, and dissemination of SARS‐CoV‐2 variants. Viral genomic data enable the identification of mutations of interest, reconstruction of transmission dynamics, and support for evidence‐based public health interventions [7]. In Senegal, genomic sequencing capacity was strengthened early in the pandemic, notably at the Institut Pasteur de Dakar, enabling the generation and analysis of several thousand SARS‐CoV‐2 genomes and the identification of circulating lineages. However, surveillance coverage remained heterogeneous across regions, limiting a comprehensive understanding of spatiotemporal viral circulation at the national scale [2, 8].

Against this backdrop, we leveraged one of the largest longitudinal SARS‐CoV‐2 genomic datasets generated in Senegal to provide an integrated national view of viral evolution and spread from March 2020 to September 2024. Beyond describing wave‐by‐wave lineage replacement, this work quantifies the following: (i) the timing and diversity of variant and Omicron sublineage emergence, (ii) the number and likely geographic sources of repeated international introductions for major Omicron sublineages, and (iii) intranational dissemination patterns between Senegalese regions using phylogenetic trait reconstruction. We also describe the genome‐wide mutational landscape across major genes, highlighting recurrent substitutions in relation to known functional and antigenic determinants. By integrating spatiotemporal lineage dynamics, introduction inference, and mutation profiling, this study provides evidence to strengthen Senegal's genomic surveillance, reduce geographic sampling gaps, and better incorporate sequence‐informed insights into routine public health decision‐making and preparedness for future respiratory epidemics.

## 2 Materials and Methods

### 2.1 Genomic Data and Metadata

A retrospective analysis of SARS‐CoV‐2 genomic data was conducted using sequences deposited in the GISAID database between 2020 and 2024. The study focused on SARS‐CoV‐2 genomes from Senegal generated and submitted by the Institut Pasteur de Dakar (IPD). All selected sequences were associated with epidemiological metadata, including at minimum the sampling date and geographic origin.

Senegalese sequences were retrieved using geographic filtering and further restricted to genomes submitted by IPD. All sequences meeting these criteria were included for descriptive analyses across the full study period, including temporal lineage dynamics (all lineages) and nationwide geographic distribution. Because Omicron represented the most densely sampled VOC and the period with the richest within‐country diversity, formal phylogenetic and phylogeographic reconstructions were subsequently focused on Omicron sublineages. Quality control filtering was also applied to retain only high‐quality, near‐complete genomes (> 90% genome coverage). Sequences with low coverage, an excessive proportion of ambiguous bases (> 10% Ns), or substantial missing data were excluded from downstream analyses.

To place Senegalese sequences within a global evolutionary context, variant‐specific reference datasets were assembled. For each lineage, the earliest and latest sampling dates observed in Senegal were used as temporal bounds for selecting contemporaneous global reference sequences from GISAID. Global sequences were subsampled using Augur [9], stratified by country and sampling date, to ensure balanced geographic and temporal representation.

### 2.2 Sequence Alignment and Phylogenetic Inference

After describing nationwide lineage turnover across all variants, phylogenetic and phylogeographic analyses were focused on the VOC Omicron, which represented the most densely sampled lineage during the study period and enabled robust inference of introduction and dissemination patterns.

Genome sequences were aligned using Nextalign (v2.14.0). Terminal regions with a high density of gaps were masked using Aliview [10] (approximately 150–200 base pairs at the 5′ and 3′ ends) to minimize phylogenetic noise.

Maximum likelihood phylogenies were inferred in IQ‐TREE (v2.1.4‐beta) using a GTR substitution model with empirical base frequencies (+F), a proportion of invariant sites (+I), and a discrete gamma distribution with four rate categories (Γ4), with 1000 ultrafast bootstrap replicates [11]. Time‐scaled phylogenies were subsequently estimated using TreeTime (v0.9.2), applying a fixed molecular clock rate of 7.0 × 10^−4^ substitutions per site per year with a standard deviation of 3.5 × 10^−4^ as defined in [12].

For visualization and computational efficiency, datasets were subsampled using Augur [9], limiting the total number of sequences to 500 per analysis while retaining all Senegalese genomes, aiming to maintain temporal and geographic representativeness of background diversity.

### 2.3 Inference of Viral Introductions and Phylogeographic Analyses

Viral introduction events were inferred separately for major VOC Omicron sublineages (including BA.1, BA.2, BA.4, BA.5, and BQ.1) using a discrete phylogeographic framework. For each sublineage, an independent time‐calibrated phylogenetic tree was reconstructed, and geographic location was modeled as a discrete trait evolving along each tree. Migration events between Senegal and international locations were estimated using the discrete “mugration” model implemented in TreeTime [12].

To investigate viral spread at the national scale, an expanded dataset including all circulating variants was constructed. Regional phylogeographic analyses were restricted to sequences with complete and unambiguous regional metadata to ensure reliable inference of interregional transmission events. This approach enabled the characterization of SARS‐CoV‐2 dissemination patterns across Senegal, independent of viral lineage.

Geographic transition events were quantified using a custom Python script developed by Eduan Wilkinson and colleagues, based on TreeTime output files [13].

### 2.4 Mutational Profiling and Genetic Diversity

Mutational diversity analyses were performed using Senegalese SARS‐CoV‐2 genomes that met strict completeness criteria (genome length > 29,000 nucleotides; coverage > 95%), across all lineages. Nucleotide substitutions and amino acid changes were identified using Nextclade [14].

Mutation data were processed and summarized using the dplyr and tidyr packages in R. Mutations were grouped by gene and amino acid position. Amino acid substitutions observed fewer than 50 times were aggregated into an “other mutations” category to emphasize dominant mutational patterns.

## 3 Results

### 3.1 Temporal and Spatial Distribution of SARS‐CoV‐2 Lineages in Senegal

**FIGURE 1:**
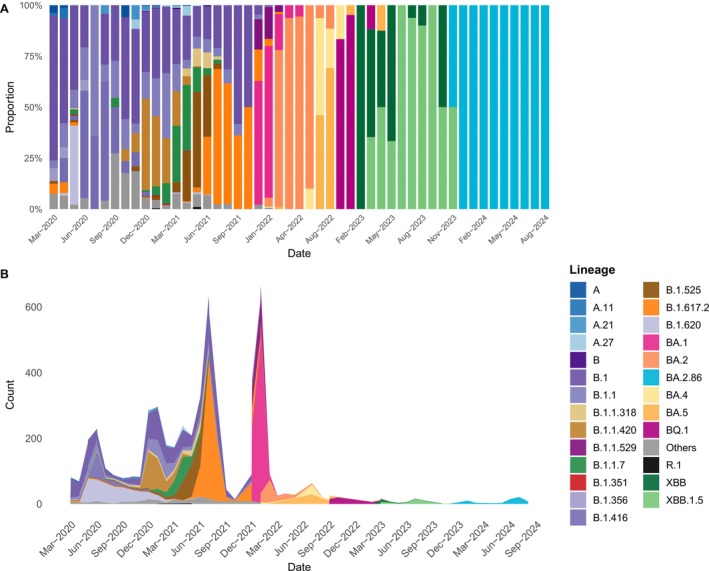
Temporal evolution of SARS‐CoV‐2 lineage diversity detected in Senegal between March 2020 and September 2024. The x‐axis represents sampling dates, while the y‐axis indicates the number of cases associated with each variant.

**FIGURE 2:**
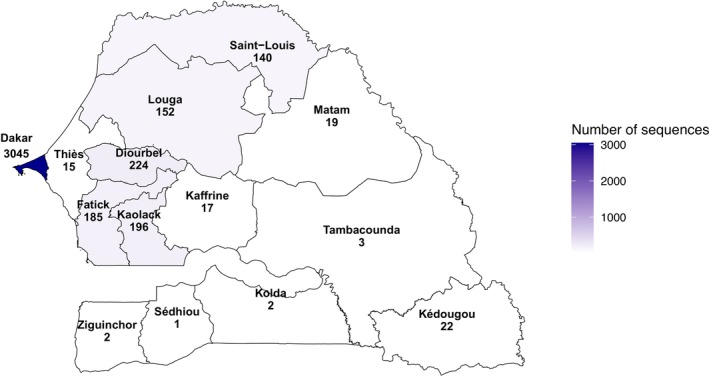
Geographic distribution of the number of SARS‐CoV‐2 sequences analyzed by region in Senegal. Each region's contribution is proportional to bar length, with Dakar showing a pronounced predominance of sequences.

This analysis was based on a dataset of 4820 SARS‐CoV‐2 genomes collected in Senegal and sequenced by the Institut Pasteur de Dakar (IPD) between March 2020 and September 2024 (Supplementary S1). These data allowed the reconstruction of the temporal dynamics of viral lineages circulating during successive epidemic waves (Figure 1) and the assessment of the geographical coverage of genomic surveillance at the national scale (Figure 2).

From March to late 2020, ancestral lineages A and B predominated, corresponding to the first epidemic wave. From late 2020 onward, successive VOCs, including Alpha (B.1.1.7), Beta (B.1.351), and Delta (B.1.617.2), were associated with increased case numbers and intensified transmission. Alpha became dominant in early 2021 before being replaced by Delta by mid‐2021. This period coincided with the highest national case burden: 49,025 cases were reported between March 2020 and May 2021, whereas 23,817 cases, nearly half that number, occurred within 3 months (June–August 2021; IPD reference laboratories), coinciding with the peak number of genomes generated through surveillance (Figure 1B). A major transition occurred at the end of 2021 with the emergence of Omicron (B.1.1.529) and successive sublineages, notably BA.1 and BA.2 in early 2022 and BA.4/BA.5 in mid‐to‐late 2022, reflecting high genetic diversity and rapid turnover (Figure 1A). From late 2022 into early 2023, BA.5‐descendant subvariants such as BQ.1 (including BQ.1.1) were detected, followed by XBB lineages and BA.2.86 in 2023–2024. Over this period, epidemic waves progressively declined in amplitude, with later subvariants such as XBB and XBB.1.5 showing more limited spread and lower epidemic intensity than Delta and early Omicron waves.

The sequences originated from multiple regions across the country, enabling an evaluation of the territorial coverage of genomic surveillance over time. A strong concentration of sequences was observed in Dakar, which alone accounted for approximately 64.7% of all generated genomes (Figure 2). Other regions showed more dispersed and moderate sampling, reflecting a more balanced but still insufficient geographical representativeness. In addition, 688 sequences (14.6%) lacked regional metadata, highlighting persistent limitations in the completeness of epidemiological metadata.

Given the sustained dominance, high sampling density, and rapid diversification of Omicron from late 2021 onward, we next focused phylogenetic and phylogeographic analyses on major Omicron sublineages to infer introduction sources and dissemination dynamics.

### 3.2 Origins and Dissemination of Omicron Sublineages Circulating in Senegal

**FIGURE 3:**
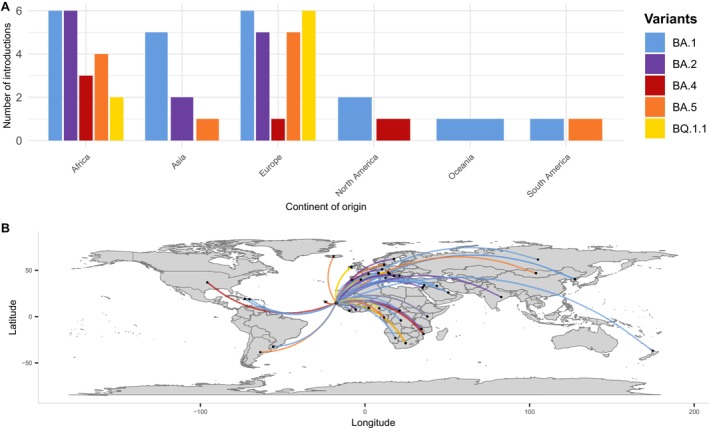
Geographic origin and introduction pathways of SARS‐CoV‐2 Omicron subvariants into Senegal. Panel A provides a histogram of the number of introduction events by continent of origin, with each subvariant represented by a specific color. Panel B displays global introduction routes to Senegal on a world map, with colored arrows linking countries of origin to Senegal.

A phylogeographic analysis of Omicron VOC sublineages circulating in Senegal was performed to infer likely geographic origins and characterize introduction dynamics, focusing on BA.1, BA.2, BA.4, BA.5, and BQ.1.1, the main Omicron lineages detected during the study period. Phylogeographic reconstruction identified multiple independent introduction events (Figure 3). Panel A summarizes introductions by continent of origin for each sublineage, while Panel B maps inferred routes linking source countries to Senegal.

BA.1 exhibited the highest number of introductions, inferred from all inhabited continents, with Africa accounting for a substantial fraction and additional contributions from Europe and Asia, plus smaller numbers from North and South America and sporadic introductions from Oceania. BA.2 also showed numerous introductions, mainly from Africa, Europe, and Asia, but with a somewhat less diverse origin spectrum than BA.1 (Figure 3). In contrast, BA.4 and BA.5 introductions were predominantly inferred from Africa, consistent with the role of southern and eastern African countries in their emergence and regional amplification, although Europe also contributed, particularly for BA.5. BQ.1.1 displayed a different profile, with most introductions inferred from Europe and fewer from Africa, reflecting its later expansion primarily in Europe before detection in Senegal.

Overall, introduction patterns varied across Omicron sublineages. Early sublineages (BA.1 and BA.2) were seeded from a broad range of continents, whereas later sublineages showed more geographically concentrated origins, dominated by Africa for BA.4/BA.5 and by Europe for BQ.1.1, underscoring shifting transcontinental transmission routes into Senegal over the course of the Omicron wave.

**FIGURE 4:**
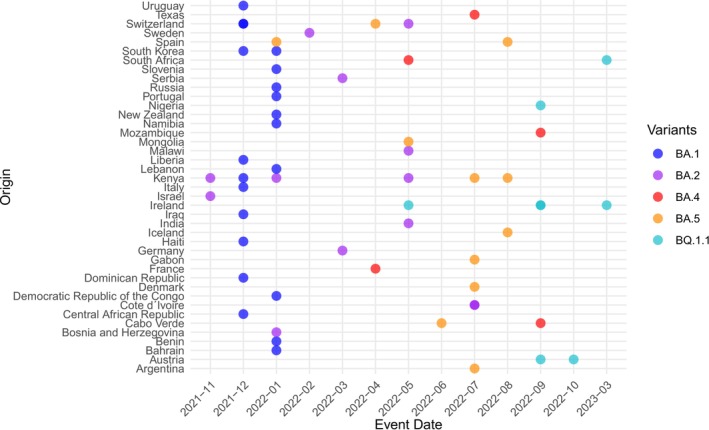
Temporal distribution of inferred Omicron introductions into Senegal (November 2021–March 2023) by country/region of origin. Each point represents one introduction event, colored by subvariant.

Figure 4 shows the temporal distribution of inferred Omicron introductions into Senegal from November 2021 to March 2023 and highlights changing geographic sources across successive subvariants. Each point represents a distinct introduction event plotted by inferred date and colored by subvariant. Introductions occurred repeatedly over time and were seeded from many countries, with no single source clearly dominating.

During the early Omicron phase (late 2021 to March 2022), introductions were mainly associated with BA.1. Events were inferred from numerous European countries (including France, Spain, Austria, Denmark, Germany, Italy, Portugal, Russia, Switzerland, and Sweden) and from multiple African countries (including Côte d'Ivoire, Democratic Republic of the Congo, Cabo Verde, Benin, Nigeria, Gabon, and South Africa), underscoring the role of intra‐African mobility. Additional early introductions were linked to Asia and Oceania (e.g., India, Lebanon, Israel, South Korea, and New Zealand), as well as the Americas, including Haiti, Argentina, and Texas. The presence of Mongolia among early BA.1 sources illustrates that some lineages reached Senegal via distant travel corridors.

In the intermediate phase (~March to late 2022), introductions increasingly involved BA.2 and later BA.4/BA.5. BA.2 sources remained diverse across Europe and Africa, with additional long‐range sources such as India, Iraq, Lebanon, Israel, Mongolia, and New Zealand. BA.4/BA.5 introductions were fewer but included events from Spain, Portugal, Switzerland, South Africa, Mozambique, Namibia, and Nigeria, with additional contributions from the Dominican Republic and Argentina.

In the late phase (late 2022 to March 2023), introductions associated with BQ.1.1 were detected from mixed sources spanning Europe, Africa, and Latin America (including Uruguay), along with an additional event from Texas. Overall, Figure 4 indicates that Senegal's Omicron epidemic was fueled by many mostly low‐frequency seeding events from diverse international and regional mobility networks, rather than repeated introductions from a single dominant source.

### 3.3 Phylogenetic Analysis of Omicron Sublineages

**FIGURE 5:**
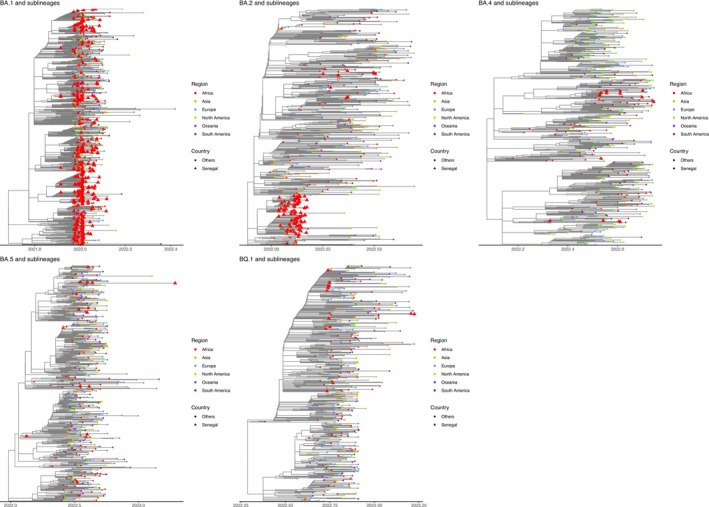
Time‐scaled phylogenetic trees of Omicron subvariants (BA.1, BA.2, BA.4, BA.5, and BQ.1.1), highlighting sequences detected in Senegal. The horizontal axis represents time. Viral sequences are shown as colored points according to their region of origin, and their shape distinguishes Senegalese sequences (red triangles).

These time‐scaled phylogenies place Senegalese Omicron genomes within global SARS‐CoV‐2 diversity and illustrate evolutionary relationships across sublineages (Figure 5). The trees show lineage‐specific patterns consistent with both Omicron temporal dynamics (BA.1, BA.2, BA.4, BA.5, and BQ.1) and heterogeneous surveillance coverage across regions.

For BA.1, Senegalese genomes are well represented and spread across multiple branches between late 2021 and early 2022, consistent with multiple introductions followed by local transmission rather than a single monophyletic cluster. BA.2 shows a similar pattern, with Senegalese sequences concentrated in several parts of the tree from early to mid‐2022; limited clustering is present, but the distribution across separated branches again supports repeated introductions.

In contrast, BA.4 and BA.5 include few Senegalese genomes, which appear sporadically and do not form well‐supported clusters, suggesting limited local amplification. These trees are dominated by European and North American genomes, reflecting higher sampling density in those regions during mid to late 2022. The low Senegalese representation may therefore reflect reduced circulation and/or lower sampling during this period.

The BQ.1 phylogeny (late 2022 to early 2023) contains very few Senegalese sequences, with Europe, Asia, and North America predominating. The lack of cohesive Senegalese clusters is consistent with sporadic introductions and minimal onward local transmission and/or limited sampling for this sublineage.

The metadata for each sequence included in these lineage‐specific analyses are provided in Supplementary S2–S6.

### 3.4 Distribution of Interregional Introductions in Senegal

**FIGURE 6:**
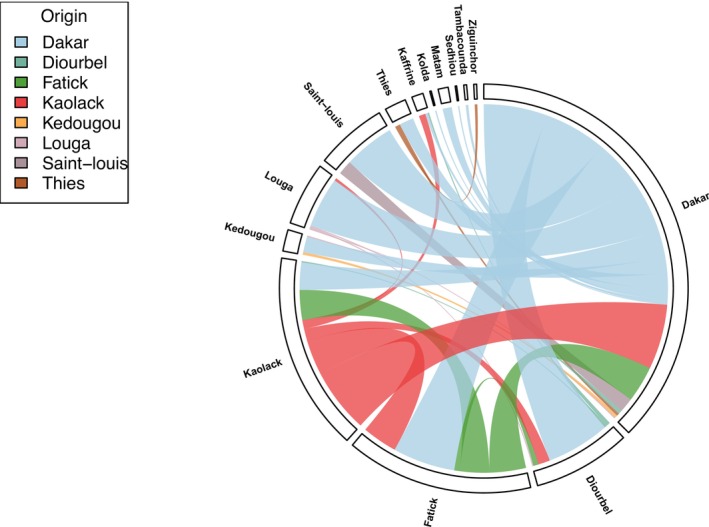
Intranational transmission flows of Omicron subvariants between regions of Senegal. Each segment represents a region of origin or destination, while colored links indicate transmission events, with colors corresponding to the region of origin.

Analysis of intranational viral lineage movements revealed a highly centralized pattern of Omicron subvariant spread within Senegal (Figure 6). The reconstructed transmission network was dominated by Dakar, which acted as the principal hub of interregional spread. In total, 97 interregional transmission events involved Dakar, either as the region of origin or destination, underscoring its central role as both a major exporter and recipient of Omicron lineages. Consistent with this, Dakar displayed extensive outgoing connections to most other regions, alongside substantial inflows from multiple parts of the country.

Secondary hubs included Kaolack and Diourbel, which contributed appreciable numbers of inferred transmission events, particularly in connection with Dakar and neighboring central regions. Strong bidirectional flows were observed between Dakar and Kaolack, as well as between Dakar and Diourbel, indicating intense reciprocal exchange along these epidemiological corridors. In contrast, more peripheral regions, particularly those in the east and south, were represented by narrower arcs and predominantly incoming, low‐volume links, suggesting that they functioned mainly as downstream recipients with limited onward transmission. Overall, the pattern of interregional spread is consistent with a hub‐and‐spoke structure centered on Dakar and supported by a small set of secondary regional hubs.

### 3.5 Mutational Profiles

**FIGURE 7:**
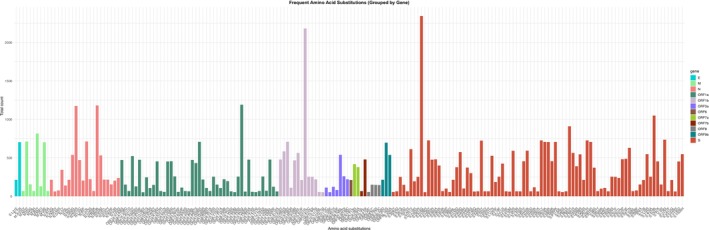
Distribution of frequent (≥ 50 occurrences) amino acid substitutions across SARS‐CoV‐2 genes. Each bar corresponds to a specific substitution, and its height indicates the total number of occurrences. Colors differentiate the viral genes involved (E, M, N, ORF1a, ORF1b, ORF3a, ORF6, ORF7a, ORF7b, ORF8, ORF9b, and S).

The mutational profile analysis was conducted using a subset of 2432 Senegalese genomes (Supplementary S7), across all lineages, meeting strict completeness criteria (genome length > 29,000 nucleotides and coverage > 95%) to identify the most frequent amino acid substitutions across viral genes (Figure 7).

The majority of observed mutations were concentrated in the Spike (S) gene, with a total of 29,967 substitutions, reflecting strong selective pressure on this key protein involved in viral entry and immune evasion. Several frequency peaks correspond to well‐characterized changes associated with increased transmissibility and immune escape, including D614G, N501Y, E484K, and multiple Omicron‐defining substitutions.

The nucleocapsid gene (N) also showed substantial mutational accumulation (7812 substitutions), suggesting considerable genetic variability in this region, potentially driven by functional adaptation or genetic drift. The ORF1a (13,379 mutations) and ORF1b (7430 mutations) regions, encoding nonstructural proteins involved in viral replication, exhibited widespread substitutions distributed along their sequences.

Additional genomic regions, including the membrane gene M (2785), ORF3a (1872), ORF7a (945), ORF7b (597), ORF8 (867), ORF9b (1603), and the envelope gene E (985), also display recurrent mutations, albeit at lower frequencies. In contrast, some regions such as ORF6 show more limited variability.

Overall, Figure 7 reveals a heterogeneous distribution of recurrent amino acid substitutions across the SARS‐CoV‐2 genome in Senegal, with especially dense accumulation in the Spike gene and in ORF1a/ORF1b, and additional gene‐specific patterns in N, M, E, and the accessory ORFs.

## 4 Conclusions

This longitudinal genomic analysis (March 2020 to September 2024) documents successive waves of SARS‐CoV‐2 lineage replacement in Senegal, culminating in sustained Omicron dominance from late 2021 onward. Early transmission was dominated by ancestral lineages A and B linked to the original strains first reported in China in late 2019 [15], followed by waves driven by more transmissible variants. Delta dominated in 2021 and caused a major epidemic wave, consistent with its high transmissibility [16]. Omicron, first detected in late 2021, triggered a rapid resurgence, largely explained by immune evasion despite reduced clinical severity compared with earlier variants [17].

Between 2022 and 2024, rapid turnover of Omicron sublineages (including BA.1, BA.2, and XBB) reflects sustained evolutionary pressure. The progressive decline in wave magnitude is consistent with increasing population immunity from vaccination and prior infections [18], reinforcing the importance of sustained genomic surveillance in Africa for early detection and public health action [19, 20].

Senegal strengthened sequencing capacity through genomic surveillance networks, yet during the study period output remained geographically heterogeneous, with 64.7% of genomes from Dakar. This likely reflects centralized sequencing and referral pathways, alongside differences in population density and healthcare access [21, 22]. The imbalance persisted despite efforts to extend capacity beyond Dakar through mobile or field laboratory deployments supporting outbreak response, including field‐based molecular testing and, in some investigations, sequencing and phylogenetic analyses [23, 24, 25, 26]. In regions such as Kedougou and Tambacounda, logistical and structural barriers likely constrained sampling and analysis, compounded by transport equipment gaps and cold‐chain limitations [19]. Persistent data‐system weaknesses were also evident, with missing regional metadata for 14.6% of sequences, underscoring the need for standardized data‐entry procedures and improved digital traceability and linkage between laboratory and epidemiological systems [13, 19].

Phylogeographic analyses indicate complex Omicron introduction and circulation dynamics. As introductions were inferred using discrete ancestral‐state reconstruction, inferred origins represent parsimonious, data‐supported explanations rather than definitive travel histories. Most inferred introductions originated from Africa and Europe, consistent with strong air travel, commercial and migratory links [27]. Dakar functioned as a key international entry point and dissemination hub, similar to other major mobility nodes such as Addis Ababa and Dubai [28]. BA.1 showed multiple origins (more than 15 countries), reflecting rapid global spread after travel resumed in late 2021 [27]. Repeated entry of genetically distinct lineages and minimum introduction estimates given incomplete sampling align with broader patterns of continual emergence of transmissible and immune‐evasive sublineages [29, 30, 31]. Within Senegal, inferred interregional flows highlight Dakar's role in internal spread, with connectivity to Kaolack, Diourbel, and Fatick, consistent with mobility‐driven transmission along transport and economic corridors [8, 32]. These findings support strengthening surveillance at entry points and across all regions to improve detection and preparedness, including for underserved settings [8, 29].

Across Omicron, Senegal's early response captured substantial BA.1 and BA.2 diversity, consistent with rapid community transmission and patterns reported in South Africa, and with trends in southern Africa and Europe [33, 34, 35]. Later sublineages (BA.4/BA.5) were underrepresented despite wider circulation elsewhere, potentially reflecting increasing immunity, heterogeneous vaccine coverage, and reduced sequencing intensity [36]. BQ.1/BQ.1.1 expanded globally in late 2022 [37] but were rarely detected in Senegal, plausibly due to lower circulation and reduced sampling amid declining funding and prioritization [38]. Because detection depends on surveillance intensity and continuity [37, 39], these patterns underscore the need for durable investment; sustained sequencing remains essential, consistent with WHO recommendations for long‐term genomic surveillance in low‐ and middle‐income countries [40].

Senegal's national COVID‐19 vaccination campaign was launched on 23 February 2021, initially using the Sinopharm BIBP vaccine, followed by doses received through the COVAX initiative from March 2021 onward [41]. In parallel, modelling work led by Diarra et al. provided an early Senegal‐specific framework for anticipating the impact of vaccine rollout under supply constraints [42]. The study indicated that immunization would need to be accompanied by continued public‐health measures to meaningfully reduce epidemic pressure, and that broader population coverage, rather than a strategy focused only on older or high‐risk groups, could offer greater benefit by limiting transmission and hospitalization burden. Real‐world vaccine uptake was initially limited: as of February 2022, only approximately 6% of the total population had completed a primary vaccination series, well below the national target of 20% coverage by mid‐2021 [43]. By the end of 2023, however, Senegal was reported among the few African countries achieving relatively high COVID‐19 booster‐dose coverage, approaching 47% of the total population [43].

Together, these findings suggest that vaccine‐induced immunity built up progressively but unevenly across Senegal between 2021 and 2023, while infection‐derived immunity had already begun accumulating before vaccine rollout. A national population‐based serosurvey conducted in October–November 2020 reported substantial anti‐SARS‐CoV‐2 seroprevalence after the first epidemic wave, indicating that confirmed case counts underestimated the true extent of population exposure [44]. In the context of successive epidemic waves driven by Alpha, Delta, and Omicron sublineages, this gradual layering of infection‐ and vaccine‐derived immunity may have contributed to the apparent reduction in epidemic wave intensity observed from 2022 onward in our genomic data. This interpretation is aligned with the Senegal‐specific modelling work which emphasized that epidemic control under constrained vaccine availability would require immunization to be complemented by sustained public‐health measures and broader population protection [42]. Accordingly, the declining representation of later Omicron sublineages such as BA.4, BA.5, and BQ.1 in Senegalese sequences may reflect not only reduced sequencing activity, but also a real decrease in viral circulation linked to increasing hybrid immunity. However, this conclusion should remain cautious. Vaccine uptake was spatially heterogeneous, with urban–rural disparities likely overlapping with the geographic imbalances in genomic sampling described above. In addition, vaccine hesitancy, affecting nearly 13% of the population and leading to outright refusal in another 33%, has been documented as a persistent challenge in Senegal, particularly in large cities [45]. Finally, available seroprevalence evidence remains largely limited to the early pandemic period, without longitudinal data covering the full 2021–2023 period. Therefore, interpreting the interplay between vaccination, natural immunity, and variant succession requires caution, particularly because available seroprevalence evidence is mainly early‐pandemic and not longitudinally available across the full 2021–2023 period.

Mutation profiling revealed heterogeneous evolutionary rates across genes, with the greatest accumulation in Spike, consistent with ACE2‐mediated entry and immune selection. Recurrently selected mutations included D614G [46], N501Y [47], and E484K [48]. The nucleocapsid gene also showed substantial diversity, including R203K and G204R, previously associated with higher viral RNA levels and/or transmission fitness in some settings [49]. Numerous substitutions were observed in ORF1a/ORF1b, which may affect replication function or antiviral sensitivity [50], and in M and accessory genes (ORF3a, ORF8, and ORF9b) linked to immune modulation and inflammatory responses [51], whereas more conserved regions such as ORF6 likely reflect strong functional constraint.

Strengths of this study include the large longitudinal dataset and integrated lineage tracking, time‐scaled phylogenetics, phylogeographic reconstruction, and mutation profiling. Limitations include geographic and temporal sequencing imbalances that may bias prevalence and connectivity estimates, sensitivity of discrete‐trait phylogeography to uneven local and global sampling, and incomplete metadata that constrained spatial resolution. Expanding decentralized sequencing and improving standardized metadata capture and linkage would enhance representativeness and the operational value of genomic surveillance for future epidemic threats.

## Author Contributions

**Ndeye Awa Ndiaye:** methodology, visualization, writing – original draft, software, formal analysis, data curation, supervision. **Safiétou Sankhe:** investigation, methodology, validation, writing – review and editing, formal analysis, data curation. **Mamadou Malado Jallow:** conceptualization, investigation, writing – review and editing, methodology, validation. **Mouhamed Kane:** investigation, methodology, formal analysis, writing – review and editing, data curation. **Khadidiatou Ndour:** formal analysis, software, visualization, writing – review and editing, data curation. **Amadou Diallo:** methodology, validation, visualization, software, formal analysis, data curation. **Maimouna Mbanne:** methodology, formal analysis. **Ndeye Marième Top:** methodology, validation, visualization, software, formal analysis, data curation. **Seynabou Mbaye Ba Souna Diop:** methodology, formal analysis. **Madeleine Dieng:** methodology, formal analysis. **Issa Gnasse:** investigation, data curation. **Debora Goudiaby:** investigation, data curation. **Cheikh Talla:** investigation, supervision, writing – review and editing. **Mamadou Aliou Barry:** investigation, supervision, writing – review and editing. **Oumar Faye:** investigation, writing – review and editing, supervision, resources. **Ousmane Faye:** conceptualization, funding acquisition, writing – review and editing, validation, supervision, resources, project administration. **Cheikh Loucoubar:** validation, methodology, supervision, writing – review and editing, resources. **Amadou Alpha Sall:** project administration, funding acquisition, writing – review and editing, resources. **Gamou Fall:** investigation, writing – review and editing, project administration, resources, funding acquisition. **Marie Henriette Dior Ndione:** conceptualization, investigation, writing – review and editing, supervision, data curation, validation, formal analysis. **Moussa Moïse Diagne:** conceptualization, investigation, funding acquisition, writing – review and editing, project administration, resources, supervision, data curation, methodology. **Ndongo Dia:** conceptualization, investigation, funding acquisition, writing – review and editing, validation, project administration, resources.

## Funding

This work was funded in part by the Africa Pathogen Genomics Initiative (Africa PGI), supported by the Bill & Melinda Gates Foundation (Grants INV‐018278, INV‐036413 and INV‐036379), by a direct Bill & Melinda Gates Foundation grant to Institut Pasteur de Dakar (INV‐018978), and by the National Institutes of Health through a supplement award (NIH 3U01AI151758‐02S1) as part of the Pasteur International Center for Research on Emerging Infectious Diseases (PICREID) project. Additional support was provided by the French Ministry for Europe and Foreign Affairs through the “REPAIR COVID‐19‐Africa” project and by Agence Française de Développement through the AFROSCREEN project (Grant Agreement CZZ3209), coordinated by ANRS|Maladies infectieuses émergentes in partnership with Institut Pasteur and IRD. The funders had no role in study design, data collection, analysis, decision to publish, or preparation of the manuscript.

## Ethics Statement

This work did not involve experiments on human participants or animals. The study was based solely on the analysis of viral genomic data generated as part of routine public health surveillance activities. No personal or clinical identifiable information was accessed. In accordance with institutional and national regulations, ethical approval was not required for this study.

## Conflicts of Interest

The authors declare no conflicts of interest.

## Supporting information


**Data S1:** Metadata table for Senegalese SARS‐CoV‐2 genomes included in the study.

**Data S2:** Metadata table for BA.1 genomes included in the lineage‐specific phylogenetic analysis.

**Data S3:** Metadata table for BA.2 genomes included in the lineage‐specific phylogenetic analysis.

**Data S4:** Metadata table for BA.4 genomes included in the lineage‐specific phylogenetic analysis.

**Data S5:** Metadata table for BA.5 genomes included in the lineage‐specific phylogenetic analysis.

**Data S6:** Metadata table for BQ.1 genomes included in the lineage‐specific phylogenetic analysis.

**Data S7:** Metadata table for the Senegalese SARS‐CoV‐2 genomes included in the mutational profile analysis.

## Data Availability

All genome sequences analyzed in this study were obtained from the GISAID EpiCoV database. Accession numbers and contributing laboratories are provided in the Supplementary Material. Data are available under GISAID's terms of use at https://www.gisaid.org.
